# A synthesis of concepts of resilience to inform operationalization of health systems resilience in recovery from disruptive public health events including COVID-19

**DOI:** 10.3389/fpubh.2023.1105537

**Published:** 2023-05-12

**Authors:** Geraldine McDarby, Redda Seifeldin, Yu Zhang, Saqif Mustafa, Mila Petrova, Gerard Schmets, Denis Porignon, Suraya Dalil, Sohel Saikat

**Affiliations:** Special Programme on Primary Health Care, World Health Organization, Geneva, Switzerland

**Keywords:** health systems, recovery, resilience, COVID-19, integrated approach, public health

## Abstract

This article is part of the Research Topic ‘Health Systems Recovery in the Context of COVID-19 and Protracted Conflict’

Health systems resilience has become a ubiquitous concept as countries respond to and recover from crises such as the COVID-19 pandemic, war and conflict, natural disasters, and economic stressors *inter alia*. However, the operational scope and definition of health systems resilience to inform health systems recovery and the building back better agenda have not been elaborated in the literature and discourse to date. When widely used terms and their operational definitions appear nebulous or are not consistently used, it can perpetuate misalignment between stakeholders and investments. This can hinder progress in integrated approaches such as strengthening primary health care (PHC) and the essential public health functions (EPHFs) in health and allied sectors as well as hinder progress toward key global objectives such as recovering and sustaining progress toward universal health coverage (UHC), health security, healthier populations, and the Sustainable Development Goals (SDGs). This paper represents a conceptual synthesis based on 45 documents drawn from peer-reviewed papers and gray literature sources and supplemented by unpublished data drawn from the extensive operational experience of the co-authors in the application of health systems resilience at country level. The results present a synthesis of global understanding of the concept of resilience in the context of health systems. We report on different aspects of health systems resilience and conclude by proposing a clear operational definition of health systems resilience that can be readily applied by different stakeholders to inform current global recovery and beyond.

## 1. Introduction

While the term resilience has been used within academic literature and global health discourse for some time, the specific concept of “health system resilience” did not gain prominence in academic literature before 2011, following the World Health Assembly resolution advocating building health systems resilience (1). It did not become widespread within global discourse until the Ebola outbreaks in West Africa and the excess mortality associated with disruptions to health services it caused (2–4). Since the start of the COVID-19 pandemic and the widespread health service disruptions associated with it, the concept of health system resilience has become ubiquitous, specifically with respect to its contribution to health security, Universal Health Coverage (UHC), and health system strengthening (5). By highlighting the mismatch between traditional health system monitoring including the UHC and global health security indices and the ability to maintain essential services in the context of a shock event, COVID-19 has demonstrated the ineffectiveness of existing approaches to strengthen health systems and promote their resilience (6). This failure to adequately consider and apply the requirements for resilience in health system planning is a factor in the persistence of foundational gaps in health systems and their continuing susceptibility to shock events despite continuing health sector investment, as seen in countries irrespective of their income groups.

A lack of clarity around the operationalization of resilience in health systems has also contributed to the global failure to build resilient health systems, by hindering effective advocacy and support to countries in building and measuring resilience. As countries and global institutions look to recovery, there is an urgent need to move beyond the conceptual, and focus efforts and resources on operationalizing resilience to ensure recovery efforts build resilience into “systems for health”^1^ and enable effective action on evolving public health challenges. This study aims to bring clarity to the concept of health systems resilience and its application, presenting a synthesis of global understanding and unpacking the key requirements for operationalization, to ensure the promotion of sustainable recovery.

## 2. Materials and methods

This paper represents a synthesis of key conceptual issues concerning health system resilience and proposes three practical areas of focus to build health systems resilience. The practical proposals draw on findings from the conceptual synthesis and critical gaps identified; and the co-authors’ considerable operational experience in health systems strengthening for resilience and recovery at country level, primarily within South Sudan, Iran, Thailand, Ireland, Liberia, and Ethiopia.

The documents included in the conceptual synthesis were initially drawn from a rapid literature review on health systems resilience in disruptive emergencies conducted in 2017 and updated in 2020. The timeline of these reviews was limited to post 2013 in order to capture literature on significant recent public health events such as Ebola and the early stages of COVID-19 (5, 7). Both reviews served to underpin WHO technical products^2^ on health systems resilience (8–10). Details of the approaches to these reviews are contained in the documents (5, 7). In summary, the core literature searches were conducted in PubMed, with supplementary searches of the websites of major organizations working in global health, including United Nations International Strategy for Disaster Reduction (UNISDR), United Nations International Children’s Fund (UNICEF), the Organisation for Economic Co-operation and Development (OECD), the Rockefeller Foundation, the UK’s former Department for International Development (DFID), Oxfam, the European Commission, United States Agency for International Development (USAID), World Health Organization (WHO) including Regional Offices and Headquarters, etc. Publications were limited by English language.

These searches were supplemented in September 2022 with a highly focused search in PubMed, aiming to identify publications which explored health systems resilience as a *concept* (theory, model, etc.). The final search string used was “resilien*” [title] AND (“concept” [Title] OR “theor*” [Title] OR “concept formation” [MeSH Terms]. Out of 274 articles retrieved, 98 underwent screening with a further 12 included in the analysis.

A total of 81 documents were reviewed as full text, with data extracted from 45; 33 from previous reviews and 12 from the updated focused search. The most common reasons for exclusion included insufficient articulation of definitional and/or conceptual issues around health systems resilience or a lack of a specific focus on resilience as it relates to health systems. Data were systematically extracted into Excel (Supplementary material Table S1). Documents were reviewed in order of perceived relevance with heavy conceptual saturation reached early in the process, with indications of saturation as early as paper 10 (11).

Findings from the conceptual synthesis were complemented with considerations arising from the co-authors’ considerable operational experience in health systems resilience and recovery. The latter has been accumulated through the implementation of a number of country level projects including the Tackling Deadly Diseases in Africa Program (TDDAP), an ongoing multi-year project on building health systems resilience funded by the Korea International Cooperation Agency (KOICA) in Ethiopia and Liberia and the pioneering of a strategic approach to the essential public health functions (EPHFs) in Ireland (2022) as well as the collaborative development of a number of technical products in support of health systems resilience including a Health Systems Resilience toolkit (8), the Primary Health Care Monitoring and Evaluation Framework (10), and a Health Systems Resilience Indicators Package (9).

## 3. Results

Results are organized in three parts. First we discuss three key thematic areas that emerged from the document review: (1) the evolution of the concept of health systems resilience; (2) definitions and attributes of health systems resilience; and (3) the operationalization of the concept, or the translation of the conceptual into tangible, measurable actions. The synthesis of ideas at this high level demonstrated that while there are a variety of disciplinary perspectives, terminology and specific considerations within the literature, there is also meaningful consensus that can form the basis of practical action. Drawing on this consensus, we then propose three key required areas of focus to foster health systems resilience: (1) embedding consideration of resilience within health system strengthening efforts; (2) ensuring the systematic capture of learning within health systems and the translation of that learning into practice; and (3) ensuring health systems have a public health orientation, such as through operationalizing the essential public health functions. The identification of these areas to promote resilience draws from critical analysis of the available literature, including gaps, in conjunction with the field experience of the co-authors in strengthening health system resilience when recovering from both acute and chronic shocks and stressors. Finally, an operational definition of health system resilience is proposed that supports the translation of the concept of resilience into tangible and measurable actions within health systems.

### 3.1. Evolution of the concept of health systems resilience

The concept of resilience as applied to systems generally emerged from the physical sciences literature in the 1970s as the ability of a system to absorb change and disturbance while still maintaining the same relationships between variables (12, 13). The promotion of absorption, adaptation, and transformation as key resilience strategies emerged from ecological literature shortly thereafter, with the strategy employed depending on the size and duration of the impact (13, 14). These three key themes remain central to health systems resilience whether they are presented as strategies, capacities, levels, or dimensions (14–16). The concept of health systems resilience has been influenced by different thematic approaches as well as global experience with public health emergencies (PHEs). Early conceptualizations presented it as the opposite of system vulnerability, which represents a mix of political, social, economic, health, cultural, and other determinant factors (17). There was an initial focus on the maintenance of infrastructure, functionality of health facilities, and continued service delivery, with this evolving to encompass what has been described as “software,” including social networks and workforce motivation (5, 18). Community resilience as a contributor to health system resilience has been increasingly reflected since the Ebola outbreaks in West Africa as has the contribution and even the agency of the individuals within the system, to overall system resilience (14). Experience with the outbreaks reemphasized the centrality of the maintenance of quality in health services and the link between health system resilience and health system strengthening (14, 19). Although the link between a lack of resilience and weak public health capacities was identified following experience with Ebola, the recognition of the strong relationship between the two has only become widespread due to the prolonged and significant disruptions associated with COVID-19 globally (12, 20). Experience with the COVID-19 pandemic has also brought the focus back to the inherent interconnectedness between multiple complex systems apparent within the concept of vulnerability, i.e., the social, economic, and political systems in which health systems are embedded (21–24). More recently resilience has been associated with recovery, transformation, the building back better agenda, and with health system strengthening more broadly (15, 25–29).

The type of shocks and stressors dominating the literature has also shifted in response to global experience with PHEs. While there is a differentiation between chronic events such as repeated reform, insufficient funding and human resources within the literature, and acute events such as natural or man-made disasters, they share the underlying principle of a disruption, which may vary in size, onset, and nature. Interestingly, response to slow onset or chronic challenges, or what has more recently been become known as “everyday resilience,” predominated the literature prior to 2011, when the focus shifted to natural disasters (5). Everyday resilience emphasizes the resources available to individuals within the system to support the daily provision of services (14). Infectious diseases have dominated the discourse since the Ebola crisis, with migration becoming more prominent since 2017 in response to mass displacements (13, 14, 30). The idea of everyday resilience has also reasserted itself within global discourse in recent times reflecting the chronicity of health system challenges that often exacerbate the impact of larger or more acute stressors like COVID-19 on the health system (18, 27, 29).

### 3.2. Definitions of health systems resilience

Explicit definitions of “health systems resilience” were sparse before Kruk’s widely cited definition from 2015; “the capacity of health actors, institutions and populations to prepare for and effectively respond to crises, maintain core functions when a crisis hits, and informed by lessons learned during a crisis, reorganise if conditions require it.” While this definition recognizes health systems as complex adaptive systems with both a reactive capacity to react to disturbances and a proactive capacity to anticipate and prepare for shocks and stressors, it fails to explicitly recognize prevention or recovery (2, 31, 32). Despite these limitations, this definition or variations on it have been central to the development of research in health systems resilience since, with the central focus being the ability to effectively manage change while maintaining essential services.

While enriching the discussion and understanding, differing perspectives have contributed to conceptual ambiguity with different authors presenting the same or similar concepts in different ways, i.e., absorption, adaptation, and transformation are presented as strategies, capabilities or levels by different authors and resilience itself presented as an outcome and a process (8, 20, 25, 26). Despite this lack of clarity, there is broad thematic agreement around what constitutes health systems resilience with a number of key themes consistently reflected, often presented as capacities. These include prevention and preparedness, response, maintenance of core or essential services or functions, and recovery (4). The majority of the definitions are focused on response, which is often equated with the strategies of absorption, adaptation and transformation, taken from the physical science literature or similar strategies or capacities seen as attributed to or promoting resilience (6, 18, 21). Despite the focus on response within definitions, there is a recognition within the accompanying narratives that resilience entails proactive and continuous action rather than just the reaction to a crisis (18). Learning, and its relationship with health system transformation and reconfiguration, is a key element of much discourse though it is often overlooked within definitions and research (7, 16, 20). Recovery is also explicitly mentioned in many formal definitions of health systems resilience, though there is a paucity of examination or measurement of the recovery aspect of health systems resilience (2, 12, 30, 31). The delivery of core or essential services or functions in all contexts is central to the demonstration of resilience, and exclusively measured during shock events, with consideration for quality (infection prevention and control; patient safety; occupational health) becoming more prevalent post Ebola (2, 11, 14).

### 3.3. Operationalization of health systems resilience

There has been broad agreement around the key attributes of a resilient health system (22, 33, 34). These are the core and interconnected features or characteristics that allow resilient health systems to prepare, respond, recover, and transform in response to shocks or stressors. The attributes most frequently cited include awareness, mobilization, self-regulation, integration, diversity, and transformation (Box 1) (23, 29, 30). Despite this convergence, there has been a relative failure to decisively move beyond the attributes to tangible and measurable actions, with critics blaming the lack of conceptual clarity for this failing. While this may certainly be a factor, health systems resilience is also complex and cross cutting with a diffusion of responsibility and accountability. Because health systems resilience is essentially everyone’s business, it becomes nobody’s business, with a lack of supporting institutional structures and a lack of targeted funding (18, 30).

BOX 1 Commonly recognized resilience attributes (2, 29).

**Table:** 

awareness	the recognition of health system capacities and risks including population health needs assessment, mapping of health system assets, and mapping and modeling potential health risks
mobilization	the ability to mobilize and coordinate resources and support including functional mechanisms to support communication and engagement between health system levels and partners including allied sectors and mechanisms for resource sharing between various stakeholders
self-regulation	making required decisions in response to threats, including the technical capacities required to identify and isolate threats, management mechanisms to support the direct targeting of resources toward identified threats and the identification of additional capacities to support response and the maintenance of services when required
integration	integration between health systems strengthening and health security and preparedness including the necessary training to recognize emergency events and activate the appropriate plans at service delivery levels across all providers and integrated surveillance systems for priority risks and threats to health
diversity	providing the range of individual and population-based services required to meet population need including the provision of agreed essential packages of services with minimization of physical, financial and social barriers and the training necessary to recognize uncommon events when they occur
transformation	identifying and applying lessons including the presence of protocols to monitor the changing performance of the health system during shock events and guidance on comprehensive recovery planning based on sector-wide assessment

Multiple frameworks have been applied within academic literature to demonstrate, measure, and classify resilience strategies, attributes, and capacities, with no single framework gaining prominence and no agreement regarding how to measure health systems resilience, although a number of “resilience indices” have been proposed (29, 35). While different, many frameworks are grounded within the WHOs health systems frameworks, using the health system building blocks as the unit of analysis or at least as a starting point (21, 24, 36, 37). Despite the prevalence of the WHO framework, the academic literature maintains a strong focus on governance, workforce and health service delivery rather than taking an integrated approach to the health system (38). A number of frameworks recognize health system strengthening as a prerequisite to the development of resilience and the resilience attributes as foundational elements of the health system (15, 17, 31). While the various frameworks present different perspectives, the majority do not stray far from the original resilience strategies of absorption, adaptation and transformation and tend to include at least some of the commonly recognized attributes.

### 3.4. Key requirements to support health systems resilience

The remainder of the results represent an expansion of the consensus on health systems resilience summarized above informed by critical reflection on the literature and considerations drawn from the co-authors experience of operationalizing health systems resilience. This seeks to address a critical block in the operationalization of health systems resilience by suggesting practical actions to be taken by policy and decision-makers working toward building health systems resilience across three key areas: integrated and resilience-focused health systems strengthening, systematic learning systems, and a system wide public health orientation. These are then incorporated into a working operational definition and aligned with existing health systems resilience indicators.

#### 3.4.1. Integrated and resilience-focused health systems strengthening

Resilience is built over time, ideally before response is required, and should be continually developed and enhanced with experiences from all contexts. While resilience is not limited to emergency response, much of what we know about resilience is taken from this context as resilience, or its lack, is most easily demonstrated during shock events (18). This has had an impact on how resilience is viewed, measured, and implemented, with a tendency to focus primarily on the delivery of health services and the development of specific emergency response capacities rather than broader health system strengthening or what has been coined “inherent system resilience” (29). While emergency response capacities are necessary to address the direct effects of system shocks, inherent system resilience is required to address the indirect effects, by supporting the daily provision of services and enabling acute response in tandem. The strengthening of existing and foundational health system elements recognizes health systems as the basis for the daily provision of services while also providing capacities that can be leveraged whenever needed to address a range of shocks and stressors, acute or chronic (Box 2) (29, 39). If not previously addressed, this requires explicit focus from early in recovery to identify and address underlying weaknesses in these foundational elements that may have contributed to the impact of the shock (40).

BOX 2 The legacy of an integrated approach to health systems (8).

At the start of the Syrian refugee crisis, Lebanon had no clear policy to address the health needs of the displaced Syrian population. The Ministry of Public Health (MPH) provided immunization and primary health care services through existing structures while international donor agencies created parallel systems, leading to fragmentation and poor coordination of the health systems response to the crisis. The MPH called for an integrated approach to planning, financing and service delivery by embedding refugee health care within the national health system. A steering committee led by the ministry and including all international and local partners, guided the response. This was supplemented by targeted recruitment to primary health care, dispensaries and public hospitals to strengthen surveillance and emergency response capacities while catering to the health needs of the refugee population. This alignment and targeting of all available resources toward strengthening existing delivery structures highlights the systems legacy made possible by an integrated approach.

Despite the focus on health service delivery, international experience has demonstrated that we need to strengthen governance and leadership, ensure adequate and sustainable financing, improve health information and surveillance systems, and strengthen human resources management and capacity (31). In short, we need a systems approach that embeds consideration of resilience within the health system building blocks as routine practice as well as in times of crisis and recovery (Table 1). This must start with high level commitment as this drives the legislative and policy environments and ultimately determines resourcing (18)(Box 3).

**Table 1:** Example of integrated and systems-based approach to health system strengthening for resilience with implications for recovery.

Health system building block	Examples of resilience-focused actions	Examples of implications for recovery and transformation efforts
Leadership and governance for resilience	Relevant authorities are enabled and provided with necessary mandate and resources to implement multisectoral national policy and strategy for protecting and maintaining population health and essential services at all administrative levels Existence of a multisectoral coordination mechanism /platform to ensure coherent actions and multisectoral accountability for public health	Establishing and strengthening dedicated institutional capacity for resilience can ensure policies, plans and regulations mandate systematic learning and application and follow-up of lessons identified. Enables the leveraging of political momentum generated during response to make policy changes that promote recovery and resilience
Resilience-focused financing	Financing models that support UHC and PHC with EPHFs and ensure proportionate investment in public health capacities Financing mechanisms that promote rapid access to funding to ensure services, workforce and supply chains in all contexts	Ensures engagement with vulnerable populations during acute response and recovery to identify and meet individual and population health needs Ensures sustainable financing for the maintenance of essential individual and population-based services in all contexts with essential social protection.
Population focused quality and accessible individual and population health services	Services oriented to identified population health needs Recognition and strengthening of PHC for UHC and essential public health functions and services encompassing emergency preparedness and response	Promotes efficient use of available resources in all contexts Strengthening primary care to deliver essential public health services can reduce the dependence on hospitals and improve community participation.
Agile and adaptable workforce	Ensure sufficient number, balanced geographical distribution and training of workforce to ensure quality and service maintenance in all contexts Consideration of surge capacities and or redeployment within training	Using recovery as platform to address gaps and improve health workforce competencies based on lessons identified Supports workforce competence and wellbeing during response and recovery
Integrated and comprehensive surveillance and monitoring of health threats, status and services	Integrated collection, analysis and interpretation of surveillance and health status and health system monitoring data Data interoperability and mechanisms that support appropriate data sharing, including pre-positioning data sharing agreements and/or strengthening and updating these during recovery.	Supports identifying health system capacities and performance baseline to inform planning with clear targets for recovery and building back better. Ensures interoperability of surveillance and health information systems and sharing of data to support decision-making
Equitable and rational access to medicines and technologies in all contexts	Equity considerations in national health service planning, delivery, and implementation Monitoring of use of medicines and technologies with specific reference to access and equity	Supports prioritization of older adults, vulnerable and marginalized populations in the recovery and transformation agenda Allows mainstreaming and scaling up relevant response-related innovations, e.g., data, supply chain management, infrastructural innovations to support recovery

BOX 3 Integrating health systems strengthening and health security for resilience (45).

Ethiopia is promoting resilience by strengthening collaboration between the health authorities and technical teams responsible for health systems strengthening and service delivery at the ministry of health and those responsible for health security in the national public health institute. Activities include joint training, risk profiling, preparing for and responding to emergencies, and planning for health service continuity, simulation exercises, post-emergency evaluations, and the establishment of governance and coordination fora. This integrated approach has ensured that each activity draws on the interconnected inputs of all health system building blocks with multisectoral participation while enabling synergies between emergency management and health systems strengthening at all administrative and service delivery levels. This has led to better alignment between health systems strengthening and health security including the establishment of an institutional focus on health system resilience in the Ethiopian Public Health Institute and adopting resilience-focused activities in national public health activities and public health emergency management guidelines as well as the identification of health service continuity as a priority with clear representation of health system and services focused teams in the COVID-19 incident management structures.

#### 3.4.2. Systematic learning systems

**Figure 1:**
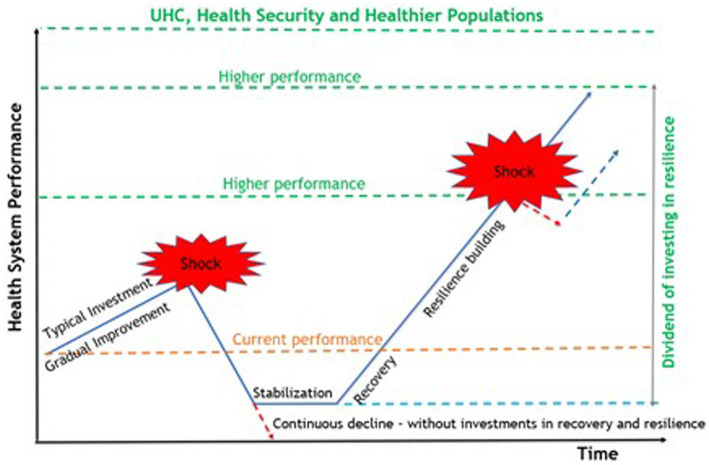
The resilience dividend.

Learning and transformation are consistently highlighted as central to the development of resilience but receive limited attention as an outcome or output, often attributed to difficulties in measurement (18, 36). Learning is key to health system strengthening and yet, the failure to implement lessons captured from prior experiences with PHEs at both national and global levels became quickly evident when COVID-19 appeared late in 2019 (38). Countries that did utilize lessons from previous PHEs, improved their health systems with strengthened public health capacities and had early success in reducing the spread of COVID-19 (Box 4) (29). Transformation is closely aligned with the goals of recovery which include rebuilding, restoration, and improvement of health system components, and relies on the ability to learn from experience (18, 36). As countries move beyond the acute phase of the pandemic the tendency is to fall back to pre-pandemic baseline levels of functioning, or back into the recognized panic and neglect cycle of emergencies (41). The systematic capture and translation of lessons identified from all contexts supports continuous improvement in services in routine times while helping to identify the new and ideal baseline for health systems in recovery to build resilience. It is this active transformation to a new sustainable baseline, above the pre-shock level but below that developed for response that supports resilience (Figure 1) (2).

BOX 4 South Korea and Vietnam: health systems learning from experience (46).

The performance of South Korea and Vietnam stood out in their response to the first wave of COVID-19. Learning from experience with Middle East respiratory syndrome, the South Korean government took a decisive and aggressive strategy to detect, screen, and isolate cases with support of surge capacities. The public was willing to follow public health advice including wearing masks and cooperating with contact tracers, and took precautionary measures.

Vietnam had both the knowledge and infrastructure to take appropriate action in early 2020 from its experiences of severe acute respiratory syndrome in 2003 and human cases of avian influenza between 2004 and 2010. For example, Vietnam took a targeted approach to testing (e.g., scaling up testing in areas with community transmission) and conducted three degrees of contact tracing for each positive case.

The need for health systems to become learning health systems that systematically generate and apply knowledge to promote continuous improvement in the behavior of the system has been long recognized (42, 43). However, learning focused activities are generally not prioritized as compared to more immediate health system pressures (42). Globally, there are systematic processes to capture lessons including Intra-action and After-action Reviews (IARs/AARs) and other post-incident reviews in virtually all countries but the majority of the recommendations remain un- or partially funded or implemented (44). The extent of implementation of lessons identified is a clear and measurable dimension of transformation, and while systematic methods to support the identification of lessons in all contexts exist, the mechanisms to ensure these lessons are incorporated into planning and budgeting cycles are often lacking.

#### 3.4.3. System wide public health orientation

Global experience with PHEs including COVID-19 has also demonstrated the historic and widespread under prioritization of public health with respect to resourcing, planning, and overall health system reform. Even within public health efforts, preventive, health promoting, and other proactive measures have been under-prioritized compared to reactive elements such as emergency response and epidemiologic capacities. This has contributed to the false perceptions that health services, including PHC, consist only of individual, and disease focused aspects of care and that public health involves only health protection and has led to the failure of health systems to fulfill their public health remit in response to current public health challenges. The failure to adequately resource public health has also prevented health systems from harnessing the benefits of preventive and promoting interventions both within and beyond the health system, to reduce the burden on secondary and tertiary care in routine emergency situations by lowering the disease burden and overall population vulnerability. As demonstrated by COVID-19 and experience with other PHEs, piecemeal or *ad hoc* development of public health capacities is insufficient and leaves populations and therefore health, economic and political systems vulnerable to shocks events (29). In the context of recovery, EPHFs and their consideration within PHC is not only critical to achieve UHC but also health security. Primary care facilities provide the first point of contact between individuals and community and national health systems, constituting a critical interface with health security and a precursor to health emergencies. In this context, the essential public health functions (EPHFs) offer a holistic and integrated approach to operationalizing public health, including emergency preparedness and response capacities, into everyday services and functions (Box 5) (30).

BOX 5 The Essential Public Health Functions (EPHFs) (47).

The EPHFs are a set of interconnected activities that provide a health system with a public health orientation. A public health orientation is advantageous as it orients health systems toward population need and governments and societies toward health and wellbeing. The EPHFs provide an integrated approach to health systems strengthening and a multisectoral approach to health focused on the wider determinants of health and equity. Investment in EPHFs strengthens core IHR (2005) and health system capacities while recognizing and strengthening the role of PHC in public health, including emergency preparedness and response and promoting multisectoral accountability. This integrates emergency preparedness and response capacities into everyday health system functioning, strengthens PHC and builds resilience.

### 3.5. Moving beyond definitions and attributes

A resilient health system can prevent, prepare for, respond to, and recover from different kinds of shocks and stressors while providing quality services. This may involve absorption, adaptation, or transformation depending on the nature, size, or duration of the shock or stressor and is expressed through key capacities which again are broadly accepted. This conceptual agreement has been sustained over time and across different thematic literatures and represents a clear consensus on what defines health systems resilience: utilizing lessons and experience to effectively prevent, prepare for, respond to, and recover from a wide variety of shocks and stressors in order to deliver high-quality individual and population health services in all contexts. Despite this consensus, recent experience with COVID-19 has demonstrated that agreement alone has been insufficient to ensure it. The effective operationalization of health system resilience must translate this consensus in definitions and attributes into the promotion of resilience through strengthening of health system foundations and public health capacities based on learning from experiences in all contexts.

### 3.6. An operational definition of resilience

**Figure 2:**
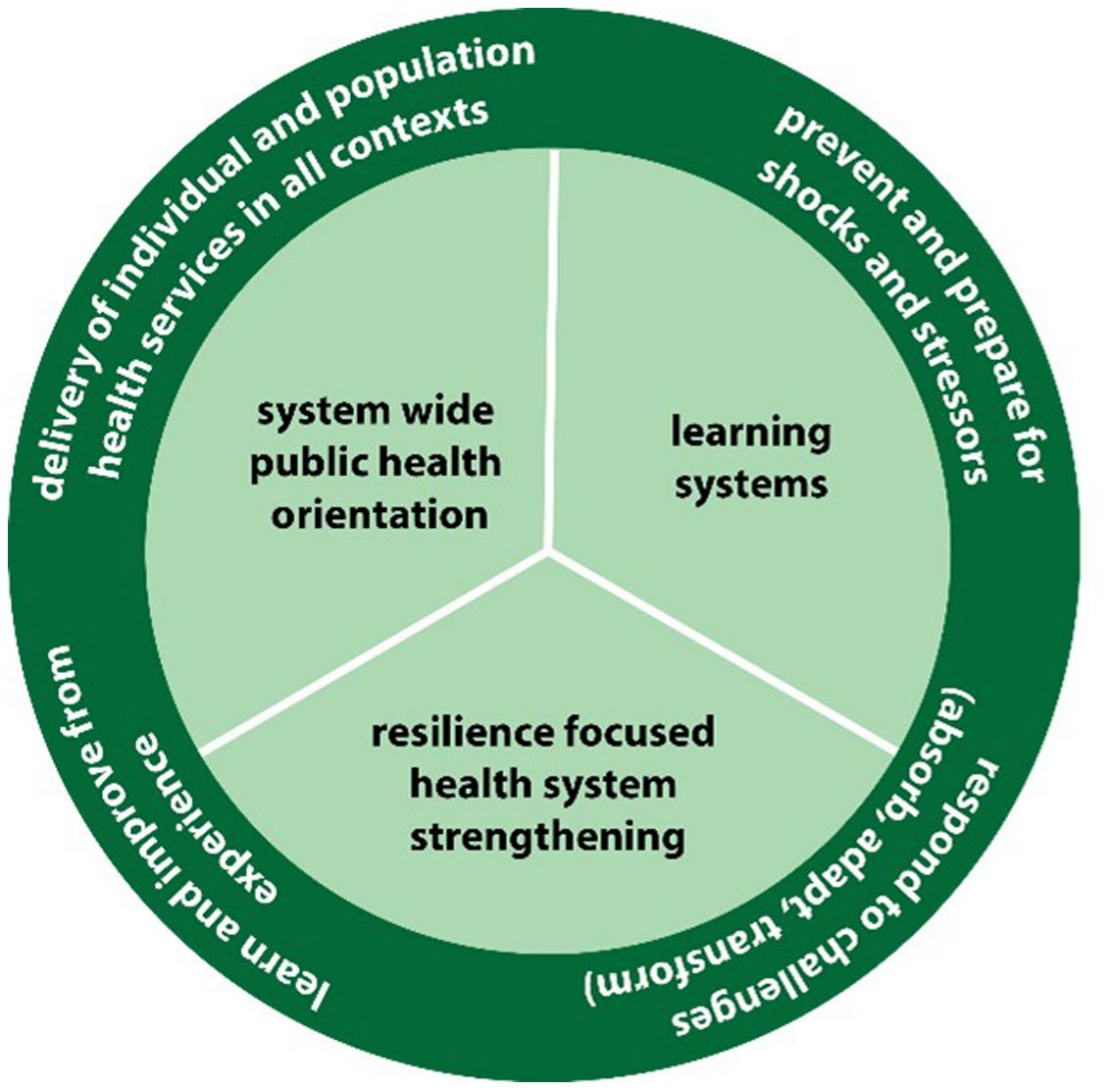
The key requirements to support resilience.

These three key requirements (Figure 2) present us with an operational definition of health systems resilience that can be applied within recovery efforts to ensure the development of health system resilience: the process of strengthening health systems to deliver quality individual and population health services oriented to population need, in all contexts by embedding considerations for resilience within all health system elements, ensuring comprehensive and integrated delivery of public health capacities, and ensuring the systematic capture and translation of lessons identified from all contexts (Figure 2). This definition recognizes that while resilience is a desired outcome, building resilience is a process dependent on three interconnected actions, which are measurable in all contexts (22). To demonstrate this, examples of indicators drawn from ongoing work on measuring health system resilience, are presented in Table 2. By making resilience measurable in all contexts, this operational definition can be used to support global advocacy toward building resilient health systems as well as enhancing recovery efforts by providing a means of embedding resilience within recovery efforts (Table 2).

**Table 2:** Key elements of resilience with example indicators for measurement in all contexts.

Key elements of resilience	Example indicator(s) (9)
Delivery of quality individual and population services oriented to need	Health service prioritization process underpinned by population health needs assessment and risk profiling Availability of a protocol or guidance for prioritization of services to be maintained in all contexts Service package for essential health services and public health functions is developed and meets criteria
Health system consideration of resilience	Proportion of health facilities including primary care that participated in any simulation exercise conducted in the last 12 months to test health system and service resilience Structures in place to support emergency management using all hazards approach Availability of a designated authority for health service/system resilience functions
Delivery of public health capacities	Strategic assessment of delivery of the EPHFs including at primary care level Existence of a national public health coordinating entity that is responsible for the integrated delivery of the EPHFs Essential public health functions are integrated into broader national health and allied sectors’ planning Health financing arrangement includes public funding of population-based services
Systematic capture and translation of lessons in all contexts	Implementation of recommendations of multi-sectoral reviews and intra and after incident assessments including a recognized budget line for activities and accountability framework Percentage of health facilities that participate in a platform to share good practices and lessons learned from emergencies from the local context and beyond Percentage of facilities that have guidance on comprehensive health system recovery planning and actions informed by situational reviews and analyses

## 4. Limitations

The synthesis was built on two rapid literature reviews that informed WHO technical products (5, 38). These reviews involved focused searches using PubMed for academic literature, and as a result, some relevant sources may have been missed. However, the synthesis was supplemented and brought up to date with a further focused review within PubMed, including a targeted search of relevant references which included a number of literature reviews of the topic. Data saturation was reached early in the updated review. The searches were additionally supplemented by searches of international organizations involved in health systems, emergency preparedness and response, and humanitarian response. While quantitative approaches to measuring resilience were identified, their scope was not sufficient to justify an independent theme and they were included within the framework section (29, 35). A detailed scoping and comparison of these was also beyond the objectives of this synthesis.

## 5. Conclusion

The current global focus on health system recovery from COVID-19 and other shocks and stressors has been intertwined with the global discourse on resilience. While recovery is an inherent aspect of resilience, like resilience it is often overlooked in health system planning and budgeting, with health systems tending to passively fall back to baseline or near baseline functions during the recovery period. This is at odds with the active improvement envisioned within definitions of recovery and contributes to the chronic ‘panic-neglect’ cycle that has dominated emergency response efforts for decades. This has been demonstrated on a large scale in the response to the current pandemic (8, 48). With global economic costs in the trillions, and far-reaching social impacts including rising inequity and poverty, it must be clear that this approach is no longer sustainable (46).

As we enter what has been called a “new age of pandemics,” current recovery efforts present us with the opportunity to learn from the past as well as an urgency to do better for the future (30, 48). The goal of recovery efforts is to build back better and transform health systems in ways that build resilience, but this process requires that the entities tasked with responsibility for the publics’ health are appointed with the authority and mandate to draw the attention and resources to target the key requirements for building resilience when establishing the new system baseline. Harnessing recovery efforts to build resilience is among the key policy recommendations of the WHO‘s unified position paper on recovery and aligned with the regional priorities set out by the Regional Committee for the Eastern Mediterranean, and while there is no doubt that investment will be required, resilience is less about the absolute availability of resources and more about the smart use of all available resources within and beyond the health sector (12, 39). Ensuring all recovery investments contribute to wider system strengthening, reorienting health systems toward more cost-effective approaches including PHC and the essential public health functions and investing in learning systems are the key investments required today to ensure health system resilience for the future.

## Data availability statement

The original contributions presented in the study are included in the article/Supplementary material, further inquiries can be directed to the corresponding author.

## Author contributions

GMD provided substantial contributions to the drafting and revising of the manuscript from conception to delivery. RS provided substantial contributions to the intellectual content of the manuscript and contributed to the revising of the manuscript and was responsible for the acquisition of significant data informing the work. YZ provided substantial contributions to the intellectual content of the manuscript and contributed to the revising of the manuscript. SM provided substantial contributions to the intellectual content of the manuscript and contributed to the revising of the manuscript. MP provided substantial contributions to the intellectual content of the manuscript and contributed to the revising of the manuscript. GS provided approval for conceptualization and design of the work including approval for publication of content. DP provided approval for publication of content. SD provided approval for conceptualisation and design of the work including approval for publication of content. SS provided overall stewardship of conception as well as the acquisition of significant data informing the work as well as contributing to manuscript revision. All authors contributed to the article and approved the submitted version.

## Conflict of interest

The authors declare that the research was conducted in the absence of any commercial or financial relationships that could be construed as a potential conflict of interest.

## Publisher’s note

All claims expressed in this article are solely those of the authors and do not necessarily represent those of their affiliated organizations, or those of the publisher, the editors and the reviewers. Any product that may be evaluated in this article, or claim that may be made by its manufacturer, is not guaranteed or endorsed by the publisher.

## Author disclaimer

The perspectives expressed in this article are those of the authors and do not necessarily represent the decisions or the policies of the World Health Organization.

## Footnotes

^1^Systems for health refers to an emerging conceptualization of health systems that moves beyond traditional approaches to measuring achievement of Universal Health Coverage (UHC) to include actions within and beyond health systems to promote healthier populations and ensure health security; World Health Organization, et al., *Systems for health: everyone has a role: flagship report of the Alliance for Health Policy and Systems Research*. 2022, World Health Organization: Geneva.

^2^WHO technical products to the documents and tools produced by the WHO in support of strengthening health and health systems in Member States.
