# Supplementary material for: A synthesis of concepts of resilience to inform operationalization of health systems resilience in recovery from disruptive public health events including COVID-19
Source: Front Public Health. 2023 May 12;11:1105537. doi: 10.3389/fpubh.2023.1105537 (PMC10213627; doi:10.3389/fpubh.2023.1105537)
Supplement: Supplementary file 1 [file Table_1.DOCX]

Supplementary Material

# A synthesis of concepts of resilience to inform operationalization of health systems resilience in recovery from disruptive public health events including COVID-19

McDarby G^1^, Seifelden R^1,^ Zhang Y^1^,Mustafa S^1^, Petrova M^1^, Schmets G^2^, Porignon D^3^ , Dalil S^4^ and Saikat S^1^

^1^Health Systems Resilience Team, Primary Health Care Special Programme, World Health Organization, Headquarters, Geneva, Switzerland

^2^ Deputy Director Primary Health Care Special Programme, World Health Organization, Headquarters, Geneva, Switzerland

^3^Country Impact Unit, Primary Health Care Special Programme, World Health Organization, Headquarters, Geneva, Switzerland

*** Correspondence:** Dr. Geraldine McDarby: mcdarbyg@who.int

## Supplementary Material

Table S.1: Data extraction matrix

| Definition used if present |
| --- |
| Is there consideration of risk reduction, mitigation or prevention within the concept?  If so which terminology is used? |
| Is preparedness considered within the conceptualization of resilience? |
| Does the conceptualization consider emergency response? |
| Does the conceptualization consider recovery? |
| Does the conceptualization consider the maintenance of core services or functions alongside response? |
| What is the focus of the concept of resilience?  Emergencies, all hazards, infectious diseases, everyday resilience, etc. |
| Are public health capacities considered within the concept of resilience? |
| Is resilience considered linear (bounce back to baseline) or as a dynamic process (transformation)? Unclear? |
| Is resilience considered an outcome (performance related and measurable) or a capacity (emergent feature; dependent on context and relationships)? Both? Unclear? |
| Is there linking to any other concepts? Sustainability, vulnerability, building back better, etc. |
| What capacities are described if any? |
| What attributes are descried, if any? |
| Is equity considered relevant or important to resilience? |
| Is resilience considered within the context of other systems (political, economic, social)? If so, which |
| Is there evidence of a systems-based approach to the consideration of resilience? |

**
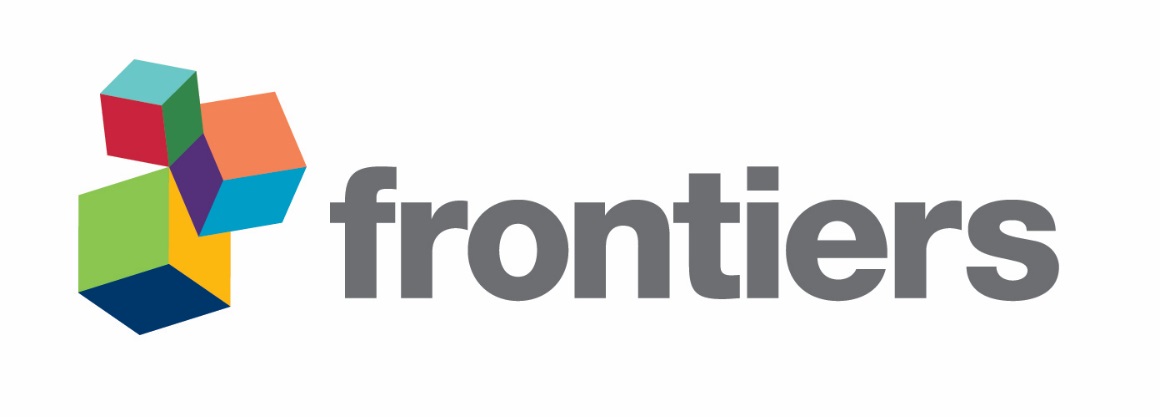
**
